# Employing Flow Cytometry to Extracellular Vesicles Sample Microvolume Analysis and Quality Control

**DOI:** 10.3389/fcell.2020.593750

**Published:** 2020-10-30

**Authors:** Joana Maia, Silvia Batista, Nuno Couto, Ana C. Gregório, Cristian Bodo, Julia Elzanowska, Maria Carolina Strano Moraes, Bruno Costa-Silva

**Affiliations:** ^1^Champalimaud Research, Champalimaud Centre for the Unknown, Lisbon, Portugal; ^2^Graduate Program in Areas of Basic and Applied Biology, University of Porto, Porto, Portugal; ^3^Digestive Unit, Champalimaud Clinical Centre, Lisbon, Portugal

**Keywords:** liquid biopsy, extracellular vesicles, exosomes, cancer flow cytometry, sample purity, microvolume, population study, longitudinal study

## Abstract

Extracellular Vesicles (EVs), membrane vesicles released by all cells, are emerging mediators of cell-cell communication. By carrying biomolecules from tissues to biofluids, EVs have attracted attention as non-invasive sources of clinical biomarkers in liquid biopsies. EVs-based liquid biopsies usually require EVs isolation before content analysis, which frequently increases sample volume requirements. We here present a Flow Cytometry (FC) strategy that does not require isolation or concentration of EVs prior to staining. By doing so, it enables population analysis of EVs in samples characterized by challenging small volumes, while reducing overall sample processing time. To illustrate its application, we performed longitudinal non-lethal population analysis of EVs in mouse plasma and in single-animal collections of murine vitreous humor. By quantifying the proportion of vesicular particles in purified and non-purified biological samples, this method also serves as a precious tool to quality control isolates of EVs purified by different protocols. Our FC strategy has an unexplored clinical potential to analyze EVs in biofluids with intrinsically limited volumes and to multiply the number of different analytes in EVs that can be studied from a single collection of biofluid.

## Graphical Abstract

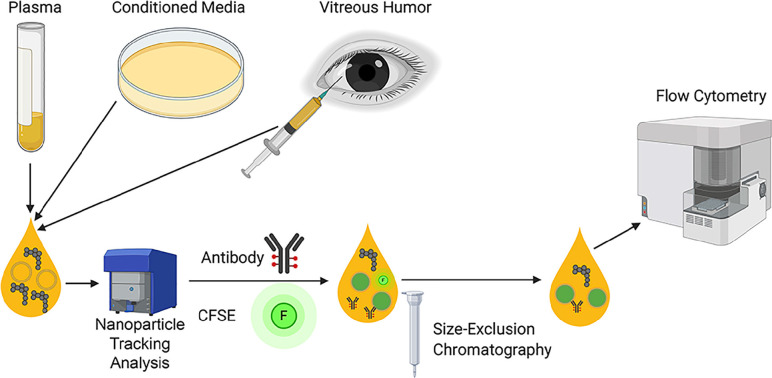

## Introduction

Extracellular vesicles (EVs), membrane vesicles released by all cells, are emerging players in cell-cell communication. In addition to differences in size and biogenesis mechanism, EVs are highly heterogeneous in molecular composition and biological properties (Groot Kormelink et al., 2016; Kowal et al., 2016; Willms et al., 2016, 2018). Their cargo includes proteins, lipids and nucleic acids, which can be transported both locally and to distant cellular targets through the peripheral circulation. As shown by us (Peinado et al., 2012; Costa-Silva et al., 2015; Hoshino et al., 2015; Rodrigues et al., 2019) and others (Kalluri, 2016), EVs can act as messengers and carriers of biomolecules in physiological and pathological scenarios (Maia et al., 2018; Stahl and Raposo, 2019).

Extracellular vesicles have been described in all body fluids (Kalluri and LeBleu, 2020), including blood (Caby et al., 2005), urine (Pisitkun et al., 2004), saliva (Ogawa et al., 2011), and vitreous humor (Ragusa et al., 2015). They serve as non-invasive sources of liquid biopsies for clinical biomarkers and allow longitudinal sampling to follow disease progression. However, high heterogeneity, which include differences in both quantity and quality of EVs, usually represent a challenge to the identification of specific EVs biomarkers in human samples. To contour some of these limitations, pre-clinical models are frequently employed, as they permit a higher degree of control over the experimental setup and sampling, and facilitate basic research related to EV composition, biogenesis and biological functions. Murine models in particular display a smaller degree of variability than clinical samples, which facilitates the discovery of biomarker candidates. However, sample volume requirements may complicate or even hamper the study of EVs in small volumes of individual collections mouse biofluids. This is mainly due to the general requirement for isolation of EVs before analysis, which inherently increases the sample volume consumption and potentially limits the number of EVs that can be harvested, thus restricting downstream analysis.

Several EV isolation methodologies are available, including separation of vesicles of similar size (e.g., by ultrafiltration, size exclusion chromatography, nanowire-based traps and deterministic lateral displacement systems), similar mass and/or density (e.g., by gradient-coupled ultracentrifugation and acoustic separation systems). It is worth noting that most of these techniques display high sample volume requirements, which can be difficult to achieve depending on the experimental context. To circumvent the volume issue, some technologies, like microfluidic lab-on-a-chip, have been developed. However, their full implementation still needs to overcome several hurdles (e.g., manual sample preparation and many non-automated chips) (Boriachek et al., 2018). Currently, there are no available methods to purify only EVs expressing endosomal features, nor consensus on markers that could be used to differentiate smaller endosomal-derived (i.e., exosomes) from larger membrane shedding-derived (i.e., Microvesicles) vesicles. Even molecules considered as markers of small endosomal EVs, such as HSP70, CD63 and CD9, have been reported to be present both in small and large EVs (Kowal et al., 2016). In the specific case of CD63, previous reports described EV isolates from several cell lines as lacking this molecule (Zhang et al., 2018). Thus, although the majority of our vesicles display exosomes features, including size (Table 1), we decided to identify them as EVs throughout the manuscript to avoid potential misidentification of our sample, following the latest MISEV’s recommendations (Thery et al., 2018). Importantly, the aim of this work is not to focus our study in endosomal EVs, but instead to present a new method that can help the study of EVs populations in general.

**TABLE 1:** Comparison of size range, sample volume and processing times between distinct isolation methods of EVs.

Sample type	Isolation method	Mean size ± standard error (nm)	Approximate sample volume for 2 × 10^9^ particles	Approximate processing time
Conditioned Medium	NP	129.3 ± 4.7 nm	137 μL	3.5 h
	ExoQ	150.6 ± 4.2 nm	287 μL	19 h
	UC-I	144.9 ± 3.3 nm	1650 μL	27 h
	UC-II	137.3 ± 3.7 nm	660 μL	26 h
	UC-III	155.1 ± 2.7 nm	480 μL	6 h
	UC-IV	162.0 ± 3.2 nm	660 μL	7 h
Plasma	NP	80.5 ± 3.8 nm	1.7 μL	3.5 h
	UC-I	140.7 ± 6.2 nm	400 μL	27 h
Vitreous humor	NP	165.5 ± 2.8 nm	5.7 μL	3.5 h

Once purified, pooled EVs can be studied for their protein (e.g., by western blotting and mass spectrometry), lipid (e.g., by mass spectrometry) and nucleic acid (e.g., by PCR, DNA and RNA sequencing) content (Contreras-Naranjo et al., 2017; Shen et al., 2017; Guo et al., 2018). Nevertheless, these methods lack information on subpopulations and molecular heterogeneity of EVs at a single-vesicle level. Technologies based on immunoaffinity-capture surfaces and beads, such as ELISA and conventional flow cytometry, have enabled the analysis of populations of EVs expressing specific antigens (Kowal et al., 2016; Guo et al., 2018). However, by capturing heterogeneous groups of EVs containing a common molecule of interest or due to resolution limitations, these methods do not allow for single-vesicle analysis of small EVs (Arenaccio and Federico, 2017; Shah et al., 2018; Zhang et al., 2018).

By tackling some of these issues, Flow Cytometry (FC) has emerged as a promising tool for thorough single-EV studies. Recently, several groups described novel strategies to stain purified EVs for FC (Morales-Kastresana et al., 2017), reported on the detection of EVs immune-captured by conventional FC (Campos-Silva et al., 2019), optimized imaging FC for analysis of EV samples (Gorgens et al., 2019) and developed new methodologies for the detection and sorting of EVs (Morales-Kastresana et al., 2019). However, existing protocols require EVs’ isolation (Nolte-’t Hoen et al., 2012; van der Vlist et al., 2012; Pospichalova et al., 2015; Groot Kormelink et al., 2016; Morales-Kastresana et al., 2017), volume concentration (Choi et al., 2019) or immunocapture of subsets of EVs (Gorgens et al., 2019) prior to staining. All these factors increase the processing time, limit the analysis of EVs to sub-populations containing biomolecules of interest and/or prevent sample analysis of small volumes and/or low numbers of EVs.

We here present a modified FC strategy that does not require isolation of EVs or concentration prior to staining, enabling the analysis of single EVs in both purified and non-purified biological samples. Determining the optimal strategy for isolating EVs for an endpoint analysis is often a critical step. Here we show the application of our FC strategy to assess the purity of isolates of EVs prepared by different protocols, thus addressing a current concern for standardization and quality control in the field of EVs (Théry et al., 2019). We also show the application of our strategy to the analysis of non-purified populations of EVs in microvolumes of individual longitudinal non-lethal collections of mouse plasma biofluids. We also demonstrate the application of our strategy to study EVs in individual mouse vitreous humor samples.

## Methods

### General Setup

The Flow Cytometry (FC) strategy presented in this manuscript is here briefly summarized and depicted in Graphical Abstract. Our general setup, starts with the NTA characterization of the biological samples. After that, the same number of particles is stained with antibody and CFSE. In order to remove of unbound CFSE and antibody, Size Exclusion Chromatography (SEC) columns are used and EVs-enriched fractions are pooled and analyzed with the Flow Cytometer Apogee A60-Micro-Plus (Apogee Flow Systems, United Kingdom).

### Cells

The C57Bl/6 murine PAN02 cell line (also identified as Panc 02, originally induced by 3-MCA, Corbett et al., 1984) was purchased from the DTP, DCTD Tumor Repository, NIH, and cultured in RPMI 1640 (Corning 15363561, NY, United States). The medium was supplemented with 10% Fetal Bovine Serum (FBS, Biowest S181BH-500, Nuaillé, France) and 1% penicillin–streptomycin (Gibco 15-140-122, United States), and maintained in a humid incubator with 5% CO_2_ at 37°C. For conditioning, cells were cultured in DMEM, High Glucose, with Glutamine, No Phenol Red (Gibco 31-053-028, United States) supplemented with 1% penicillin–streptomycin and 10% EV-depleted FBS. FBS was depleted of bovine EVs by ultracentrifugation at 100,000 *g* for 140 min. For the preparation of conditioned medium, 1 × 10^6^ PAN02 cells were seeded per 150 mm culture dish containing 20 mL of medium, and the conditioned medium was collected after 72 h of culture.

### Tumor Induction, and Plasma and Vitreous Humor Collection

All mouse work was performed in accordance with national animal experimentation guidelines (DGAV), animal protocol 0421/000/000/2018. Adult C57Bl/6 female mice (5–8 weeks old) were used for all experimental procedures. Mice were anesthetized using isoflurane 1.5–3%. Mouse vitreous humor was collected from naïve mice using 29G syringes (BD 324892, New Jersey, United States) inserted at a 45° angle into the vitreous cavity 2 mm posterior to the limbus. The volume of vitreous humor collected per mouse was in average 5 μL, where 5 μL of vitreous humor was diluted in 20 μL of 5,000 U-I-/mL heparin.

For tumor induction, a suspension of 1.5 × 10^6^ PAN02 tumor cells, resuspended in 30 μL Matrigel (Corning 354230, NY, United States), was injected intrahepatically with 29G syringes in a total of 19 animals. The experiments had a length of 14 days. Blood was collected on the day prior (Day 0), and 7 and 14 days after intrahepatic injection of PAN02 tumors. On Day 0 and Day 7 the blood was collected by submandibular bleeding via blood collection lancets, and on Day 14 it was collected by retro-orbital bleeding via heparinized glass capillary tubes. The volume of plasma collected per mouse was in average 20 μL, which were diluted in 50 μL of 5,000 U-I-/mL heparin.

### EVs’ Flow Cytometry

The A60-Micro-Plus machine (Apogee Flow Systems, United Kingdom) is equipped with three spatially separated lasers (488 nm – Position C, 405 nm – Position A and 638 nm – Position B), 7 fluorescence color detectors (525/50, LWP590, 530/30, 574/26, 590/40, 695/40, 676/36) and 3 light scatter detectors (SALS, MALS, and LALS). More details are available in Table 2.

**TABLE 2:** Apogee A60 configuration and laser power.

Channel number	Short channel name	Full channel name	Optical filter name	Laser wavelength	Laser power	PMT voltage
Ch1	405-SALS	Small Angle Light Scatter		405 nm	200 mW	400 V
Ch2	405-LALS	Large Angle Light Scatter		405 nm	200 mW	400 V
Ch3	405-Grn	Green Fluorescence	BP-525/50	405 nm	200 mW	500 V
Ch4	405-Org	Orange Fluorescence	LWP-590/35	405 nm	200 mW	500 V
Ch5	APC	Red Fluorescence	BP-676/36	638 nm	150 mW	550 V
Ch6	CFSE	Green Fluorescence	BP-525/50	488 nm	200 mW	525 V
Ch7	PE	Orange Fluorescence	BP-575/30	488 nm	200 mW	500 V
Ch8	488-Red	Red Fluorescence	BP-676/36	488 nm	200 mW	500 V
Ch9	488-DRed	Deep Red Fluorescence	LWP-740	488 nm	200 mW	500 V

**FIGURE 1:**
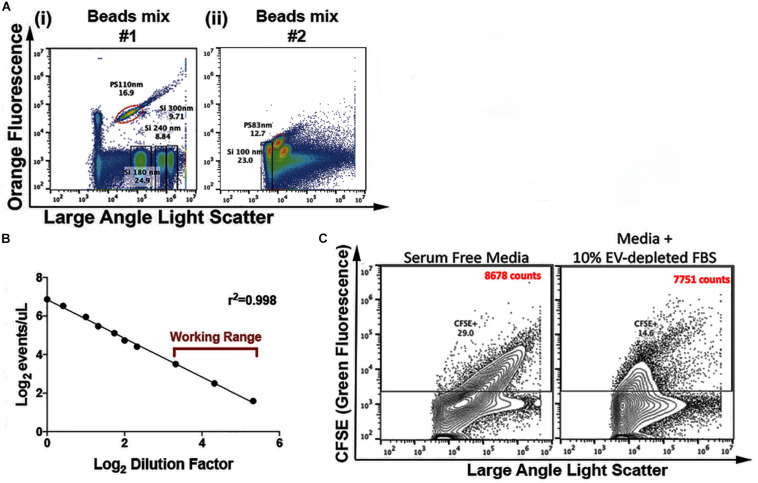
EVs’ Flow Cytometry parameters. **(A)** Flow cytometry analysis of a mix of polystyrene (PS) and silica (Si) beads. Depicted in the flow cytometric plots are: 110 nm PS, 180 nm Si, 240 nm Si, 300 nm Si (i) and 83 nm PS and 100 nm Si beads (ii) **(B)**, analysis of events/μL in relation to sample dilution factor. The utilized working range is indicated (*n* = 5 – representative plot). **(C)** Representative plots of CFSE-labeled serum-free and 10% EV-depleted FBS medium; the indicated counts (CFSE^+^) correspond to the events within the CFSE^+^ gate.

As internal controls across assays, before each FC experiment we used two commercially available mixes of beads (Apogee 1493 and Apogee 1517, Apogee Flow Systems, United Kingdom). Silica and Polystyrene beads were used as reference particles for the EV detection. Using the previous described settings (Table 2), it is possible to detect particles that scatter light similarly to 100 nm silica (SiO_2_) beads (Figures 1Ai,ii). In this ApogeeMix, SiO_2_ beads have a refractive index (η) (η = 1.42–1.43) close to that of EVs (η∼1.39) (Chandler et al., 2011; van der Pol et al., 2012).

To identify event coincidence and swarming regime (Libregts et al., 2018) in the experimental settings, serial dilutions of purified EVs were performed. The working range was set in the linear region within the operational range indicated by the Apogee A60-Micro-Plus manufacturer (maximum of 3,000 events/second) (Figure 1B). Before loading, samples were diluted in filtered PBS to bring their concentration within the working range of the equipment (maximum of 3,000 events/second). All samples were run at a flow rate of 1.5 μL/min using a 405 nm – LALS threshold of 70. For the control samples, equivalent running times is the stopping criteria utilized (e.g., 200 s). Thus, in these cases control samples are captured for equivalent amounts of time in order to fairly compare the number of positive counts between the different conditions. For the population analysis experiments, the stopping criteria utilized is the number of events acquired, so samples are acquired until a minimum of 250,000 events is reached. The 405 nm – LALS PMT was monitored and always maintained below 0.35. This number is an indicator of noise, since it displays the amount of background current, which is a function of the amount of background light reaching the photomultiplier.

For the control experiments (Figure 1C and Supplementary Figures S1A,D, S4), equivalent running times was the stopping criteria utilized. Thus, in these cases control samples were captured for equivalent amounts of time in order to fairly compare the number of positive counts between the different conditions. For the population analysis experiments depicted, the stopping criteria utilized was the number of events acquired, so samples were acquired until a minimum of 250,000 events was reached. In all experimental settings, the data was not pre-gated based on the scatter signals. The acquired data was exported and analyzed with FlowJo software v10.4.2 (FlowJo LLC, United States). All flow cytometry files are available at “flowrepository.org” under the Repository ID FR-FCM-Z2EH. The link for access to the files is:

flowrepository.org/id/RvFrS5PMa2qxxbQeAKuwxsgluJLyTs fcivorJMOOPQ0rMi6fCvkTxsQmIMXgv54S

### Validation of the EVs Staining Protocol

As a simple approach to normalize the input for our staining protocol, all samples were analyzed for particle concentration and size distribution by the NS300 Nanoparticle Tracking Analysis (NTA) system with red laser (638 nm) (NanoSight – Malvern Panalytical, United Kingdom). Samples were pre-diluted in filtered PBS to achieve a concentration within the range for optimal NTA analysis. Video acquisitions were performed using a camera level of 16 and a threshold between 5 and 7. Five to nine videos of 30 s were captured per sample. Analysis of particle concentration per mL and size distribution were performed with the NTA software v3.4.

For staining, 2 × 10^9^ particles of non-purified (NP) sample or purified EVs were mixed with 40 μL of filtered PBS containing 0.4 μg of anti-CD9 conjugated to phycoerythin (PE) (Thermo Fisher Scientific LABC 12-0091-81, Massachusetts, United States) and incubated for 1 h at 37° C. Samples were incubated with Carboxyfluorescein Diacetate Succinimidyl Ester (CFSE – Thermo Fisher Scientific LTI C34554, MA, United States) in a final concentration of 25.6 μM for 90 min at 37°C. The staining with CFSE was done after the addition of the antibody to ensure that the dye would not interfere with the antibody staining. For removal of unbound CFSE and antibody, Size Exclusion Chromatography (SEC) columns (iZON qEV original columns SP1, United Kingdom) were used. Samples containing unstained or stained EVs, and appropriate controls, were diluted up to 500 μL with filtered PBS and processed by qEV following manufacturer’s instructions. EVs-enriched fractions #7, #8, and #9 (500 μL each) were then pooled and retrieved for analysis with the Flow Cytometer Apogee A60-Micro-Plus (Apogee Flow Systems, United Kingdom).

The FC strategy here presented relies on the staining of vesicular particles with CFSE, as previously described (Pospichalova et al., 2015; Morales-Kastresana et al., 2017; Mastoridis et al., 2018). A subset of the experiments shown here was performed with conditioned medium, in which cells were grown in medium containing EVs-depleted FBS. To control for the presence of serum-derived vesicles in our samples, serum-free medium and medium containing 10% of EVs-depleted FBS were stained with CFSE and analyzed using our standard settings, with flow cytometry data being acquired for similar periods of time. CFSE^+^ events count in medium containing 10% of EVs-depleted FBS were as low as those found in serum-free medium, about 3% of the number of events acquired in EV samples from conditioned medium (Figure 1C).

For all subsequent analyses, quadrant thresholds were established with unstained and single-stained EVs. Vesicle-free controls containing CFSE, anti-CD9, and both CFSE and anti-CD9 are also shown. Control samples were captured using equal time periods, similar to the acquisition times of corresponding samples containing EVs (Supplementary Figure S1A). Increments in the concentration of CFSE didn’t increase the proportion of CFSE^+^ EVs (Supplementary Figure S1B), suggesting that our experimental conditions for CFSE staining are optimal. To ensure that Anti-CD9 staining was performed in optimal experimental conditions, incremental concentrations of Anti-CD9 were tested (Supplementary Figure S1C). The working amount of Anti-CD9 was set at 0.4 μg per staining reaction. To certify that the observed CFSE^+^ events were indeed vesicles, purified EVs were pre-incubated with 2% NP-40 for 1 h at room temperature and analyzed using our FC strategy. A reduction of 90% of CFSE^+^ events was found upon detergent treatment, confirming that most of the observed CFSE^+^ events were indeed EVs (Supplementary Figure S1D).

### NP-40 Treatment

Extracellular vesicles were lysed by incubation with 2% NP-40 (Thermo Fisher Scientific 85124, MA, United States) for 1 h at room temperature, and then stained with CFSE and analyzed by our FC strategy, as described above.

### Calculation of MESF Values

**FIGURE 2:**
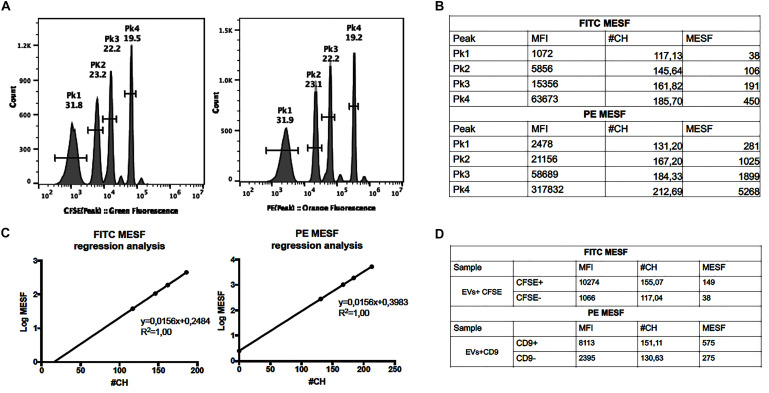
Assessment of MESF values. **(A)** Histograms of the RCP-05-5 beads in the FITC and PE channel. **(B)** The FITC and PE Median Fluorescence Intensity (MFI) of each peak was measured and converted to Relative Channel Number (#CH), and the MESF values calculated (using template provided by manufacturer). **(C)** Calibration graphs and linear regression where the MFI is plotted in the *x*-axis and log MESF is plotted in the *y*-axis. **(D)** Calculation of FITC and PE MESF values for EVs samples stained with CFSE or Anti-CD9.

Assessment of standardized unit molecules of equivalent soluble fluorochrome (MESF) values allows for cross comparisons between different instruments and laboratories (Schwartz et al., 2004; Wang and Hoffman, 2017). In this work, MESF for PE and FITC were then used to determine the fluorescence intensity of anti-CD9 and CFSE staining, respectively. The MESF were calculated for PE and CFSE using SPERO^TM^ Rainbow Beads Calibration Particles (Spherotech RCP-05-5, United States) according to instructions provided by the manufacturer. The MESF values were measured, with the same acquisition settings applied for all the assays and using a set of Rainbow Beads containing 4 bead populations with known equivalents of FITC molecules and 4 bead populations with known equivalents of PE molecules (Figure 2A). After data collection, the Median Fluorescence intensity (MFI) of these peaks was converted to Relative Channel Number (#CH) using the formula [Relative Channel# (#CH) = (R/4)log_10_(MFI), where R is the resolution]. Then, the #CH values of the Rainbow beads were plotted against log MESF values and a linear regression was performed (Figures 2B,C). The MESF values for unknown samples was calculated using the equation corresponding to this linear regression. Therefore, this regression allowed for the calculation of MESF values of our experimental controls. In our experimental setting the gates for CFSE^+^ and CD9^+^ were defined such that the CFSE^–^ and CD9^–^ populations displayed approximately 517 and 371 MESF, respectively. The CFSE^+^ population was approximately 10501 MESF whereas the PE^+^ population (CD9) was approximately 1441 MESF (Figure 2D).

### Purification and Characterization of EVs

**FIGURE 3:**
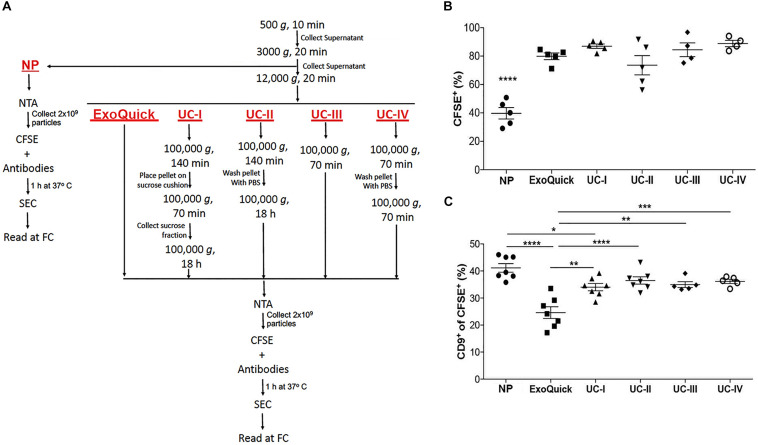
Comparison of isolation methods of EVs. **(A)** Flow chart for sample processing by ultracentrifugation (UC), ExoQuick and for analysis of non-purified (NP) samples. **(B)** Proportion of CFSE^+^ EVs in non-purified conditioned medium (NP) vs. conditioned medium purified by ExoQuick^®^ or four distinct protocols of ultracentrifugation (UC-I-IV), as indicated in the Figure 3A. ****P < 0.0001 by ANOVA, with Tukey’s post-test. **(C)** Proportion of CD9^+^ events within CFSE^+^ EVs in NP, and medium purified by ExoQuick and ultracentrifugation (UC-I-IV). ****P < 0.0001, ***P < 0.001, **P < 0.01, *P < 0.05 by ANOVA, with Tukey’s post-test. All data are represented as mean ±SEM.

Mouse blood (3.5 mL) and supernatant fraction of conditioned medium (80 mL) were centrifuged at 500 *g* for 10 min. The collected supernatant was then centrifuged at 3,000 *g* for 20 min (these samples are from now on referred to as “non-purified” – NP – samples), followed by another centrifugation at 12,000 *g* for 20 min. After these initial steps, purification of EVs was performed in plasma by UC-I (bellow) and in conditioned medium according to one of the following protocols (Figure 3A):

- (Exoquick) ExoQuick^®^ commercial kit (System Biosciences EXOTC10A-1, Palo Alto, CA, United States), following manufacturer’s instructions;
- (UC-I) 100,000 *g* for 140 min, followed by pellet resuspension in 14.5 mL of filtered Phosphate-Buffered Saline (PBS, Corning 15313581, NY, United States). This sample was pipetted on top of a 4 mL sucrose cushion (D_2_O containing 1.2 g of protease-free sucrose and 96 mg of Tris base adjusted to pH 7.4), and centrifuged at 100,000 *g* for 70 min. The fraction of interest (4 mL) was aspirated with a 18G needle and taken to a final volume of 20 mL with filtered PBS. The sample was then centrifuged at 100,000 *g* for 18 h. The EV-containing pellet was resuspended in filtered PBS;
- (UC-II) 100,000 *g* for 140 min. Pellet was washed with filtered PBS and centrifuged at 100,000 *g* for 18 h. The EV-containing pellet was resuspended in filtered PBS;
- (UC-III) 100,000 *g* for 70 min, followed by resuspension of the EV-containing pellet in filtered PBS;
- (UC-IV) 100,000 *g* for 70 min. Pellet was washed with PBS and again centrifuged at 100,000 *g* for another 70 min. The EV-containing pellet was resuspended in filtered PBS.

For the non-purified samples (NP) of conditioned medium (100 μL), mouse plasma (20 μL in 50 μL of 5,000 U-I-/mL heparin) and vitreous humor (5 μL diluted in 20 μL of 5,000 U-I-/mL heparin), a first centrifugation was performed at 500 *g* for 10 min. The supernatant was collected and a second centrifugation step was performed at 3,000 *g* for 20 min. The supernatant after this second centrifugation corresponds to the non-purified sample.

All solutions used (PBS and sucrose cushion) were sterile (0.22 μm membrane-filtered). The ultracentrifugation (UC) steps were performed in refrigerated conditions (4°C) with rotors 50.4Ti or 70Ti (Beckman-Coulter, California, United States).

### Statistical Analysis

Error bars in graphical data represent means ± SEM. Statistical significance was determined using either a two-tailed Student’s *t*-test or an ANOVA test. A *P*-value under 0.05 was considered statistically significant. Statistical analyses were performed using the GraphPad Prism software (GraphPad Version 7, CA, United States). No statistical method was used to predetermine sample size. The experiments were not randomized, and the investigators were not blinded to allocation during experiments and outcome assessment.

## Results

### Quantification of Populations of EVs in Conditioned Medium

As remarkably noted by the MISEV2018 guidelines, in a growing field as the EV research, methodologies’ standardization is of the utmost importance (Thery et al., 2018). Since there is no single optimal separation method, it’s critical for EV studies to choose the best suitable strategy for isolating EVs. Therefore, this decision still poses as a significant challenge in the field, where various EV isolation techniques are available.

In the light of these facts, our FC strategy was first employed in the quality and efficiency assessments of different EV isolation protocols. In order to perform such comparison, the proportion of vesicular (CFSE^+^) and non-vesicular (CFSE^–^) particles in samples prepared by different protocols was analyzed.

The quantification of vesicles in conditioned medium (NP) from native tumor cells by our FC strategy showed that ∼40% of particles were CFSE^+^ (Figure 3B and Supplementary Figure S2A). On the other hand, samples purified by sucrose cushion-coupled differential ultracentrifugation (UC-I), considered a high-specificity method, contained ∼85% of CFSE^+^ vesicles. Different EVs isolation protocols by ultracentrifugation may utilize different combinations of washing and density flotation steps. Therefore, the impact of these variations in the proportion of CFSE^+^ vesicles in the final isolate was tested (Figure 3B). With the exception of samples ultracentrifuged overnight without a prior density flotation step (UC-II), in which the proportion of vesicular structures was ∼70%, all other ultracentrifugation-derived preparations contained ∼85% of vesicular structures. As in ultracentrifugation-based methods, ExoQuick^®^ generated samples containing ∼80% of vesicular structures (Figure 3B). These findings indicate in the samples purified by the selected methods the majority of particles present were CFSE^+^ EVs.

The impact of isolation methods in populations of EVs was also tested. Based on previous reports indicating expression of CD9 in EVs of endosomal origin (Willms et al., 2016), CFSE^+^ populations containing this tetraspanin were measured. Although displaying a slight reduction in UC-I, the proportion of CD9^+^ in CFSE^+^ vesicles was comparable between non-purified samples (NP) and those obtained after isolation by ultracentrifugation. On the other hand, samples processed by ExoQuick^®^ contained lower levels of CD9^+^ within CFSE^+^ vesicles (Figures 3C and Supplementary Figure S2B). This suggests that isolation protocols indeed have an impact on the quality of the sample analyzed, since they may select for subpopulations of EVs.

### Quantification of Populations of EVs in Plasma

In several studies of EVs as liquid biopsies, the sample volume demanded is difficult to achieve due to the nature of the experimental conditions. Therefore, a methodology that relies on non-purified biological samples may enable novel experimental approaches. Taking that into account, our FC strategy was next used to quantify EVs in purified and non-purified plasma.

**FIGURE 4:**
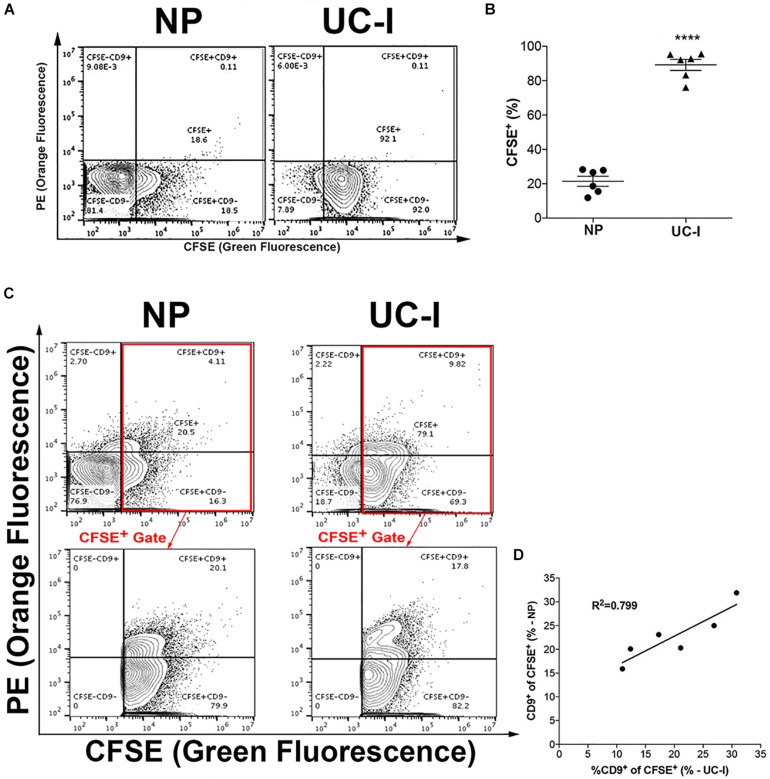
Characterization of EVs in plasma. **(A)** Plots are representative of CFSE^+^labeled particles from non-purified (NP) plasma, or from plasma purified by ultracentrifugation (UC-I). **(B)** Proportion of CFSE^+^ EVs in non-purified plasma (NP) vs. plasma purified by sucrose cushion-coupled ultracentrifugation (UC - I). ****P < 0.0001 differences vs. NP by two-tailed *t*-test. Data are represented as mean ± SEM. **(C)** Representative plots of particles labeled with CFSE and anti-CD9 from NP and UC-I plasma; the lower panels indicate the CD9^+^ and CD9^–^ events within CFSE^+^ particles. **(D)** Correlation analysis of CD9^+^ events within CFSE^+^ EVs in samples before (NP) and after purification (UC-I).

The proportion of CFSE^+^ vesicles in purified and non-purified plasma was measured. While ∼90% of particles in purified plasma samples (UC-I) were CFSE^+^ vesicles, this percentage decreased to approximately 20% in non-purified plasma (NP) (Figures 4A,B). Next, the population distribution of CD9 in EVs from mouse plasma was analyzed. Between 15–32% of CFSE^+^ plasma EVs were CD9^+^. Importantly, this level was comparable between samples that had been purified by UC-I and in native plasma samples (Figures 4C,D).

### Longitudinal Population Studies of EVs in Microvolumes of Plasma

After confirming that the results between samples of purified and non-purified plasma were comparable (Figure 4D), our FC strategy was employed in a longitudinal sampling scheme. This longitudinal experimental design was chosen due to its similarity to the ones desired for studies of EVs as liquid biopsies, e.g., to follow disease progression or treatment efficacy.

**FIGURE 5:**
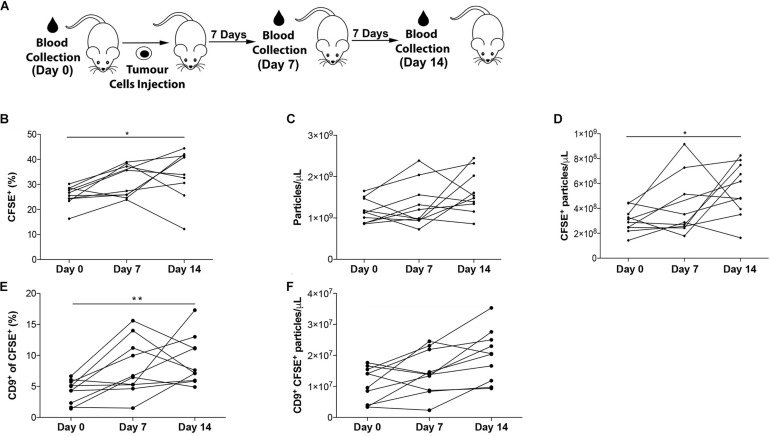
Longitudinal analysis of EVs in plasma. **(A)** Experimental setup. **(B)** Proportion of CFSE^+^ EVs in non-purified plasma of mice prior (Day 0), and 7 and 14 days after intrahepatic injection of PAN02 tumors. **(C)** Concentration of total particles per microliter of plasma, by NanoSight. **(D)** Concentration of CFSE^+^ EVs per microliter of plasma. **(E)** Proportion of CD9^+^ events within CFSE^+^ EVs. **(F)** Concentration of CD9^+^ CFSE^+^ EVs per microliter of plasma. **P < 0.01, *P < 0.05, differences vs. Day 0 by ANOVA, with Kruskal–Wallis post-test. All data are represented as mean ±SEM.

The applicability of our FC strategy to longitudinal measurements of populations of EVs was tested in microvolumes of non-purified mouse plasma. The presence of tumor cells is expected to modify the plasmatic levels of EVs during the course of the disease. Therefore, plasma from tumor-bearing mice was collected at different time points (Bebelman et al., 2018). Specifically, blood was collected from each mouse prior to intrahepatic injection of PAN02 pancreatic cancer cells (Day 0), and one (Day 7) and 2 weeks (Day 14) post-injection, as depicted in Figure 5A. In this longitudinal analysis of plasma from 10 animals, the proportion of CFSE^+^ events significantly increased in eight of the experimental subjects between days 0–7, in five between days 7–14 and in nine between days 0–14 (Figure 5B and Supplementary Figure S3A). When analyzed in relation to the total concentration of particles present in each time point measured by Nanosight (Figure 5C), the concentration of CFSE^+^ events *per* μL increased in 6 animals between days 0–7 and days 7–14, and in all animals between days 0–14 (Figure 5D).

CD9^+^ populations were measured using the experimental settings already described. The proportion of CD9^+^ within CFSE^+^ events increased in 8 animals between days 0–7, in 7 animals between days 7–14 and in 9 animals between days 0–14 (Figure 5E and Supplementary Figure S3B). Similarly, when analyzing this data in relation to the concentration of particles present in plasma (Figure 5C), an increase of CFSE^+^CD9^+^ events *per* μL in 6 animals between days 0–7 and in 9 animals between days 7–14 and 0–14 (Figure 5F) was observed.

### Quantification of Populations of EVs in Vitreous Humor

**FIGURE 6:**
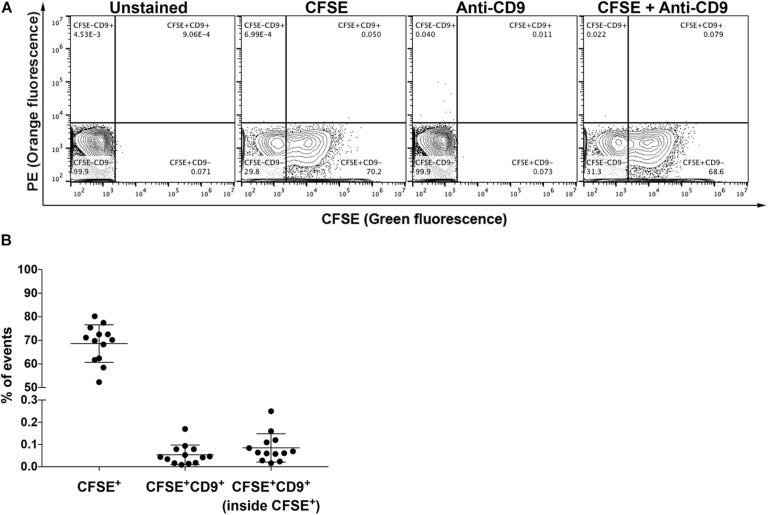
Analysis of EVs in vitreous humor. **(A)** Representative plots of unstained and stained EVs from non-purified vitreous humor from naïve mice in single-animal collections, as indicated. **(B)** Proportion of CFSE^+^ events, CFSE^+^CD9^+^ events and CD9^+^ events within CFSE^+^ EVs. All data are represented as mean ±SEM.

To further illustrate the application of our FC strategy in performing population analysis of EVs in microvolumes of samples, this workflow was used to study EVs in non-purified vitreous humor, of which 2–2.5 μL *per* mouse/*per* eye was collected. Approximately 68% of particles were CFSE^+^ vesicles (Figures 6A,B). However, the levels of CD9^+^ events in vitreous humor (<0.1% – Figure 6B) were as low as those found in control PBS containing CFSE and anti-CD9 during equivalent sample running times, which is in accordance with the literature (Murthy et al., 2014; Skeie et al., 2015; Zhao et al., 2018) as discussed below (Supplementary Figure S4).

## Discussion

### Measurement of CFSE^+^ Events as a Strategy to Assess Sample Purity

The lack of consensus regarding methods for purification of EVs remains a challenge, especially when reproducibility between different isolation modalities is desired. This limits EV use in clinical applications and is further complicated by the insufficient means to measure sample purity, notably in complex biological samples containing a mixture of vesicular and non-vesicular particles, such as plasma. Multiple strategies, such as measurement of protein:particle ratio (Webber and Clayton, 2013; Maiolo et al., 2015) and albumin (Baranyai et al., 2015; Veremeyko et al., 2018), have been proposed as quality control parameters for the preparation of EVs, particularly when low specificity isolation methods are employed (Théry et al., 2019). However, because they fail to provide population information, it is still unclear whether these metrics can accurately reflect the purity of preparations of EVs.

CFSE has to date been frequently used as a strategy to label EVs for FC applications (Pospichalova et al., 2015; Morales-Kastresana et al., 2017; Mastoridis et al., 2018; Morales-Kastresana et al., 2019). The suitability of different dyes to efficiently stain and identify EVs has already been addressed by the Jennifer Jones group (Morales-Kastresana et al., 2017). In a work published in 2017, the Jones’ group tested several dyes, including CFSE and PHK26. While PKH26 was shown to produce 100–400 nm micelles or aggregates both in the presence of EVs or alone in solution, CFSE alone did not form such aggregates. Thus, taking this into account, in our work EVs were labeled with CFSE. The data here presented supports the vesicular labeling with CFSE as a means for determining quality control of purification protocols of EVs. As shown, the majority of particles present in samples purified by high-specificity methods were CFSE^+^. The employment of ExoQuick^®^ or ultracentrifugation for enrichment of EVs prior to SEC reduced the proportion of CFSE^–^ particles. In addition, lysis of EVs with the detergent NP-40 resulted in a reduction of 90% of CFSE^+^ events. Together, these results support the usefulness of CFSE as a tool to identify vesicular particles in biofluids, and thus the suitability of our FC strategy for quality control and quantitative comparison of isolates of EVs prepared by different protocols.

This same approach was employed to validate the depletion of EVs from the FBS used in our *in vitro* studies. Previous reports suggest that traces of bovine EVs may persist in the FBS supernatant after depletion steps (Shelke et al., 2014), which could interfere in the analysis of EVs from conditioned medium. However, comparison of serum-free medium and medium containing 10% of EVs-depleted FBS showed an equally reduced proportion of CFSE^+^ events. This indicates that residual FBS-derived EVs was not high enough to impact our analysis. Nonetheless, future studies using our strategy for analysis of EVs in conditioned medium will need to account for background events on a case-by-case basis, especially when using low cell numbers, short conditioning time and/or cells producing low levels of EVs.

Results presented by others indicate that CFSE staining could not label 100% of EVs (van der Vlist et al., 2012; Pospichalova et al., 2015; de Rond et al., 2018). Thus, we reasoned that some of the CFSE^–^ events observed in our experiments could correspond to non-stained EVs. However, unlike the results presented here, in these previous studies CFSE staining was performed in cells before (van der Vlist et al., 2012) or during medium conditioning (Pospichalova et al., 2015), and CFSE concentrations were 1.6–16 times lower (van der Vlist et al., 2012; Pospichalova et al., 2015; de Rond et al., 2018) than those we used. In addition, only ∼15% of the events in samples purified by differential ultracentrifugation coupled with sucrose cushion (UC-I) were CFSE-, and treatment of EVs with the detergent NP-40 caused a reduction of 90% of CFSE^+^ events, indicating that this dye labels the majority of EVs in our experimental settings. While this may still suggest that CFSE is not capable of staining all EVs present in the sample, it is still unclear to which extent even high-purity isolation methods may provide 100% pure preparations of EVs. Although undesirable, protein aggregation may be present in antibody preparations. This is mainly due to solution conditions, such as ionic strength, pH, temperature, pressure and excipients (Manning et al., 1995), and intrinsic properties of antibodies, such as primary sequence, tertiary structure, non-symmetrical hydrophobicity and charge distributions (Liu et al., 2005; Roberts, 2007; Nishi et al., 2010). Therefore, the potential contribution of unbound antibody aggregates to CFSE^–^ Antibody^+^ events was tested. Most CFSE^–^CD9^+^ events didn’t correspond to unbound antibodies, and were reduced by EVs purification (Supplementary Figure S5). These results suggest that the CFSE^–^ events observed in the CD9^+^ population analysis correspond to non-vesicular particles of specific molecular composition with similar size as compared to EVs. Future studies, including detailed morphology and composition analysis, will be necessary to further define these CFSE^–^ non-vesicular particles.

### Comparison of Purification Methods of EVs by FC

In spite of being considered a high-recovery and low-specificity method (Théry et al., 2019), isolation based on precipitation polymers such as ExoQuick^®^ resulted in a high proportion of CFSE^+^ EVs. This agrees with studies suggesting that EVs prepared by ultracentrifugation or precipitation polymers are comparable (Helwa et al., 2017; Prendergast et al., 2018). In samples prepared by ExoQuick^®^, however, we found that the proportion of CD9^+^ CFSE^+^ events was reduced when compared to NP and other isolation methods. This suggests that, despite providing a high yield of EVs, ExoQuick^®^ may insert EV population bias. Also of concern, precipitating agents have previously been linked to potential loss of biological activity (Paolini et al., 2016) and structure (Gamez-Valero et al., 2016) of EVs. Thus, our data adds yet another parameter that should be carefully considered before selecting ExoQuick^®^ as a method of choice for the isolation of EVs.

SEC is the technique of choice for many groups interested in studying the composition and biological activity of vesicles, as it allows simple, fast and affordable isolation of EVs. As SEC is a key component of our FC strategy, the proportion of CFSE^+^ particles in our preparations was measured to access the isolation efficacy of EVs by SEC. In our experimental settings, this preparation is referred to as NP (non-purified), since SEC is used after staining, and not before as a traditional isolation method. Although considered a low recovery, high specificity method (Théry et al., 2019), conditioned medium processed by SEC contained less than 40% of CFSE^+^ vesicular particles. This was also the case for more complex samples, such as plasma and vitreous humor, in which the percentage of CFSE^+^ particles after SEC processing were, respectively, ∼20% and ∼68%. These findings agree with recent studies using comparative transmission electron microscopy, in which SEC-derived preparations displayed a lower proportion of structures resembling EVs when compared to samples derived from differential ultracentrifugation (Takov et al., 2019).

Differential ultracentrifugation is one of the most commonly used EV purification methods. To improve EV purity, most researchers combine ultracentrifugation with additional techniques following the primary step, such as the use of washing steps with saline as well as the use of density gradients (Thery et al., 2018). We found that the proportion of CFSE^+^ events and CD9^+^ events within the CFSE^+^ gate did not differ in the absence (UC-III) or presence (UC-II and UC-IV) of washing steps with PBS or when a sucrose cushion step was used (UC-I). Our results suggest that these additional steps have no major impact in sample purity. However, a more detailed characterization of the potential impact of these washing and/or separation steps in the selection of populations of EVs with specific composition (protein, sugar, lipid, and nucleic acids) will be necessary in future studies.

Our FC strategy allows for faster processing times and also substantially decreases the sample volume requirements compared to conventional EVs isolation protocols (Table 1). Thus, we consider ours to be a consistent approach to be applied to control the quality of preparations of EVs.

While we used CFSE as a general EV marker, our experimental setup was performed having CD9 not only as an illustration of the capabilities of the method, but also as an analyte of interest. Our side-by-side comparison of CFSE^+^CD9^+^ events in samples submitted (UC-I) or not (NP) to isolation by ultracentrifugation showed that both strategies are comparable when analyzing CD9^+^ EVs populations (Figures 4C,D). However, although this equivalence was true for CD9^+^ EVs, we cannot discard the possibility that other populations of EVs behave differently, specially while probably there is no validated universal EV marker that can be used as an experimental control. Every method, including differential ultracentrifugation, potentially inserts population isolation bias to EVs (as illustrated in Figure 3C). Such isolation bias, if existent, has yet to be studied and understood. Be it for future studies in the field of population of EVs, or be it for studies involving other vesicular molecules, we believe and strongly suggest that side-by-side validation of non-purified versus purified samples (as in Figures 4C,D) should be performed as a pre-validation of our approach. Thus, for every EV molecule of interest, the approach presented in our work that relies on the use of NP samples in different contexts should first be validated by a comparison study similar to the one presented in Figures 4C,D.

### Longitudinal Study of EVs in Plasma by FC

Longitudinal composition analyses can provide precious temporal information on the dynamics of EVs in physiological and pathological settings (Eitan et al., 2017; Menon et al., 2018; Wu et al., 2018). However, these studies are often difficult in microvolumes of samples, mainly due to the limited number of EVs that can be harvested in these experimental conditions (Théry et al., 2019). This constraint frequently leads to insufficient recovery of EVs (Clayton et al., 2018), unless small volume samples are pooled from multiple individuals or collections. Moreover, in studies involving small animals, the requirement for lethal bleeding in order to collect enough plasma for the effective isolation of EVs complicates the performance of longitudinal studies and increases the demand for animals, leading to higher costs, higher sample processing complexity and potential bioethical issues. By not requiring isolation of EVs prior to staining, our FC strategy allows for the analysis of both intra- and inter-individual heterogeneity in the population of interest throughout an experiment. In our studies, the proportion of CFSE^+^ EVs and of CD9^+^ events within CFSE^+^ EVs increased in the plasma of mice bearing liver metastatic pancreatic cancer lesions. Based on these results, we are currently studying the potential use of these readouts for follow-up studies of pancreatic cancer patients in the metastatic phase.

### Study of EVs in Vitreous Humor by FC

The vitreous humor is a small-volume biofluid that contains low protein content, ranging from 120 to 500 ng/μL (Chowdhury et al., 2010), which is frequently considered to arise from filtration of plasma through fenestrated capillaries of the ciliary body stroma via the iris root (Freddo et al., 1990). Besides the quantitative differences in protein content, a comparison of vitreous humor and plasma proteome revealed that only 58% of the vitreous humor proteins have also been identified in human plasma (Chowdhury et al., 2010). Consistent with this, our analysis revealed that vitreous fluid contains three times more CFSE^+^ vesicular structures when compared to plasma. Furthermore, it contained insignificant levels of CD9^+^ events, comparable to those found in control solutions with only CFSE and anti-CD9. These results are consistent with the previously reported absence of CD9 in EVs from vitreous humor (Murthy et al., 2014; Skeie et al., 2015; Zhao et al., 2018). However, it is still unknown whether the absence of CD9^+^ vesicles is a result of the filtration that occurs during the production of vitreous humor, uptake and degradation of this vesicle population by ocular cells, higher prevalence of non-endosomal EVs and/or other mechanisms. Although it is unclear to which extend ocular cells contribute to the collection of EVs found in vitreous humor, our FC strategy can be potentially used to study these vesicles both in pre-clinical and clinical settings as potential biomarkers and biological mediators of eye diseases.

## Conclusion

By allowing for the analysis of conditioned medium volumes as small as 100 μL, our FC strategy can be potentially used to characterize the heterogeneity of EVs and the differential packaging of biomolecules during the biogenesis of EVs in highly controlled small-scale in vitro systems. This approach also makes it possible to study populations of EVs from as little as ∼1 μL of plasma or vitreous humor. By doing so, our strategy represents a precious tool to identify novel physiological and pathological cell-to-cell communication systemic networks involving EVs in *in vivo* models.

In regard to the use of EVs as liquid biopsies, clinical relevance relies on experimental data that is comparable/translatable to human practice. Although *in vitro* assays are very helpful, murine models are especially relevant in this context, since they capture the complexity of human processes and often are the most advanced pre-clinical models to study the biological significance of EVs in a multicellular organism, unravel novel EV biomarkers and analyze disease stages that would be inaccessible or nonviable in direct human studies. However, in this ever-growing pre-clinical field some hurdles must be overcome. Namely, the lack of EVs isolation analytical reproducibility, which limits its clinical applicability; and the sample volume requirements, which limits the application of EVs as liquid biopsies, especially with the potential for longitudinal sampling to follow disease progression. In native clinical samples, our FC strategy has an unexplored capability to be applied in the study of populations of EVs, multiplying the number of possible different analysis from a single biofluid collection. It also has a great potential to enable the study of populations of EVs in samples with intrinsically limited volumes, such as lacrimal, vitreous humor and synovial fluids, facilitating the use of these biofluids as liquid biopsies on clinical settings.

## Data Availability Statement

The original contributions presented in the study are included in the article/Supplementary Material, further inquiries can be directed to the corresponding author.

## Ethics Statement

The animal study was reviewed and approved, being performed in accordance with national animal experimentation guidelines (DGAV), animal protocol 0421/000/000/2018.

## Author Contributions

JM designed and performed the all experiments. SB, NC, and AG contributed to the animal experimentation. CB contributed to the sample processing, EVs characterization and equipment maintenance. JE contributed with the Pan02 EVs samples for Supplementary Figure S2. MS built the experimental setup. BC-S conceived the project. All the authors wrote and reviewed the manuscript.

## Conflict of Interest

The authors declare that the research was conducted in the absence of any commercial or financial relationships that could be construed as a potential conflict of interest.

## Supplementary Material

Supplementary Figure 1 Internal controls and detergent lysis of EVs. **(A)** Representative plots of buffer containing CFSE only, anti-CD9 only, and CFSE and anti-CD9 (upper panels), and of unstained EVs, EVs stained with CFSE, EVs stained with anti-CD9, and EVs double stained with CFSE and anti-CD9 (lower panels); the indicated counts correspond to the events within the upper right quadrant (CFSE^+^CD9^+^). **(B)** Titration of CFSE staining. **(C)** Titration of Anti-CD9 staining. **(D)** Detergent lysis of EVs. The plots are representative of unstained and non-lysed EVs (EVs), CFSE-stained non-lysed EVs (EVs+CFSE), unstained EVs lysed with NP-40 (EVs+NP-40) and CFSE-stained EVs lysed with NP-40 (EVs+NP-40+CFSE); the counts of CFSE^+^ particles are indicated. The graph indicates the reduction in the percentage of CFSE^+^ EVs after lysis with NP-40. All data are represented as mean ± SEM.

Supplementary Figure 2 Different purification methods of conditioned medium samples. **(A)** Representative plots of CFSE-labeled particles from non-purified conditioned medium (NP), and from conditioned medium purified by ExoQuick or by distinct ultracentrifugation protocols (UC-I-IV). **(B)** Representative plots of particles labeled with CFSE and anti-CD9 from NP, and from conditioned medium purified by ExoQuick or by ultracentrifugation (UC-I-IV). The lower panels indicate the CD9 status within CFSE^+^ EVs.

Supplementary Figure 3 Longitudinal analysis of plasma EVs. **(A)** Representative plots of CFSE^+^labeled EVs from non-purified plasma of mice prior (Day 0), and at 7 and 14 days after intrahepatic injection of PAN02 cells. **(B**) Representative plots of particles from NP plasma double labeled with CFSE and anti-CD9; the lower panels indicate the CD9^+^ and CD9^–^ particles within CFSE^+^ EVs.

Supplementary Figure 4 CD9^+^ events in vitreous humor. Representative plots of PBS and vitreous humor double labeled with CFSE and anti-CD9. Samples were captured during 250 s. The indicated counts (CFSE^+^CD9^+^) correspond to the events within the upper right quadrant.

Supplementary Figure 5 Analysis of false-positive CD9^+^ events in conditioned medium. **(A)** Calibration curve of false-positive events. Buffer containing anti-CD9, in the same concentration used in the staining reaction and pre-cleared by SEC, was analyzed by Flow Cytometry for increasing acquisition times. **(B)** Estimate percentage of false-positive events by unbound anti-CD9 within CFSE^–^CD9^+^ in each purification context. **(C)** Proportion of CFSE^+^ (black) and CFSE^–^ (white) events within CD9^+^ events in NP samples and samples purified by ExoQuick and ultracentrifugation (UC-I-IV). ^****^*P* < 0.0001 by ANOVA, with Tukey’s post-test. All data are represented as mean ±SEM.
