# Supplementary figures and images for: Employing Flow Cytometry to Extracellular Vesicles Sample Microvolume Analysis and Quality Control
Source: Front Cell Dev Biol. 2020 Oct 30;8:593750. doi: 10.3389/fcell.2020.593750 (PMC7661467; doi:10.3389/fcell.2020.593750)

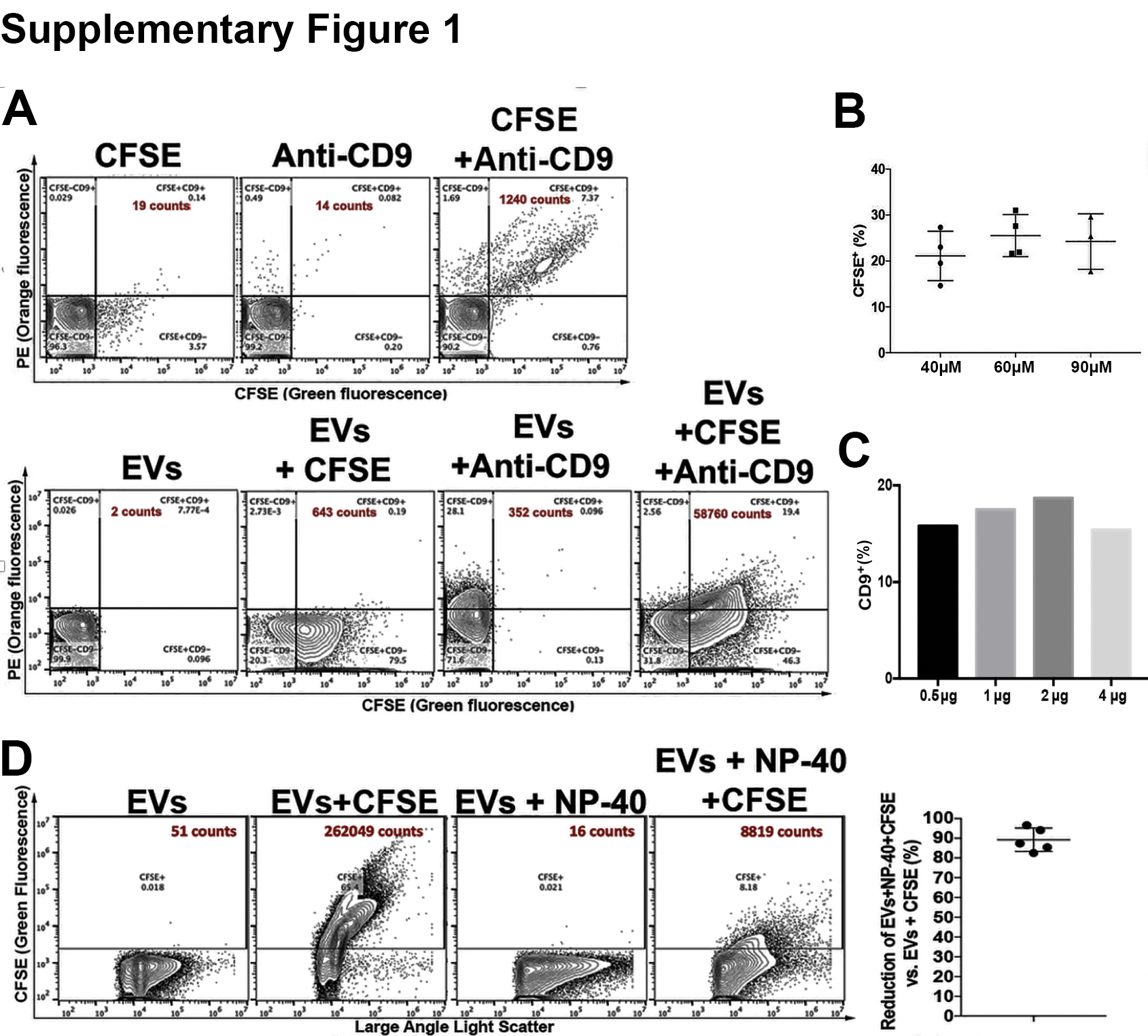

Supplement: Supplementary Figure 1 — Internal controls and detergent lysis of EVs. (A) Representative plots of buffer containing CFSE only, anti-CD9 only, and CFSE and anti-CD9 (upper panels), and of unstained EVs, EVs stained with CFSE, EVs stained with anti-CD9, and EVs double stained with CFSE and anti-CD9 (lower panels); the indicated counts correspond to the events within the upper right quadrant (CFSE+CD9+). (B) Titration of CFSE staining. (C) Titration of Anti-CD9 staining. (D) Detergent lysis of EVs. The plots are representative of unstained and non-lysed EVs (EVs), CFSE-stained non-lysed EVs (EVs+CFSE), unstained EVs lysed with NP-40 (EVs+NP-40) and CFSE-stained EVs lysed with NP-40 (EVs+NP-40+CFSE); the counts of CFSE+ particles are indicated. The graph indicates the reduction in the percentage of CFSE+ EVs after lysis with NP-40. All data are represented as mean ± SEM. [file Image_1.tif]

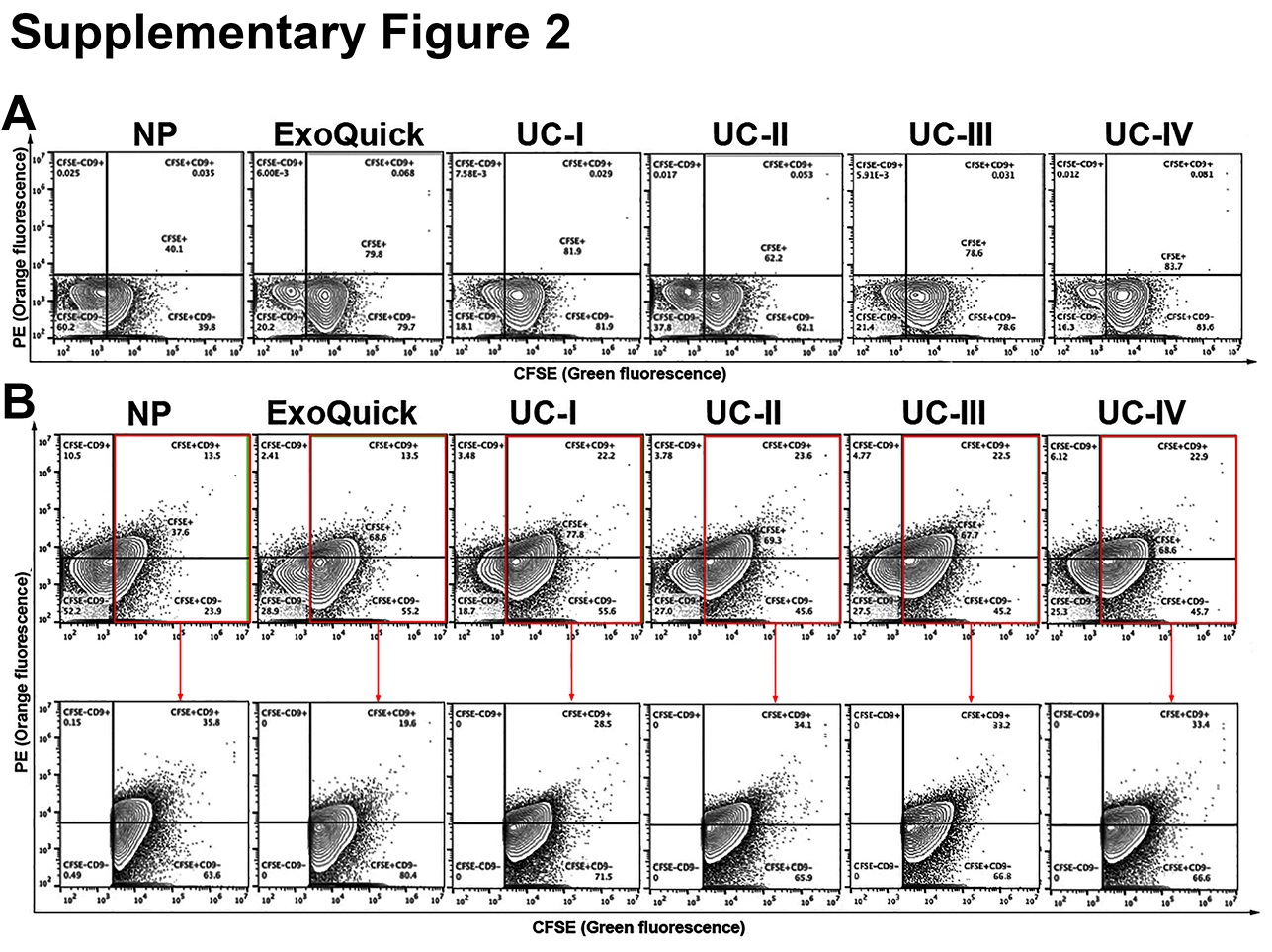

Supplement: Supplementary Figure 2 — Different purification methods of conditioned medium samples. (A) Representative plots of CFSE-labeled particles from non-purified conditioned medium (NP), and from conditioned medium purified by ExoQuick or by distinct ultracentrifugation protocols (UC-I-IV). (B) Representative plots of particles labeled with CFSE and anti-CD9 from NP, and from conditioned medium purified by ExoQuick or by ultracentrifugation (UC-I-IV). The lower panels indicate the CD9 status within CFSE+ EVs. [file Image_2.tif]

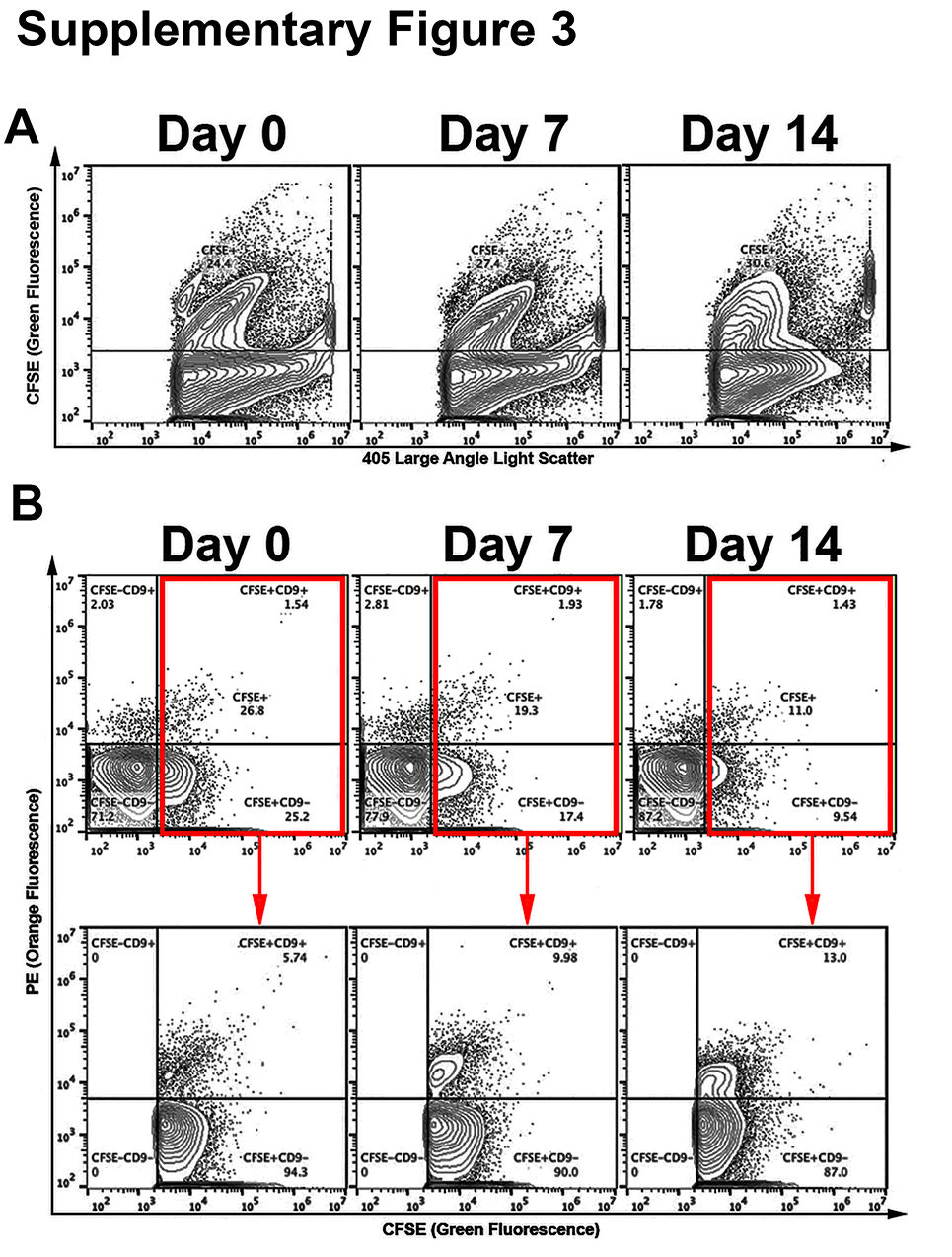

Supplement: Supplementary Figure 3 — Longitudinal analysis of plasma EVs. (A) Representative plots of CFSE+labeled EVs from non-purified plasma of mice prior (Day 0), and at 7 and 14 days after intrahepatic injection of PAN02 cells. (B) Representative plots of particles from NP plasma double labeled with CFSE and anti-CD9; the lower panels indicate the CD9+ and CD9– particles within CFSE+ EVs. [file Image_3.tif]

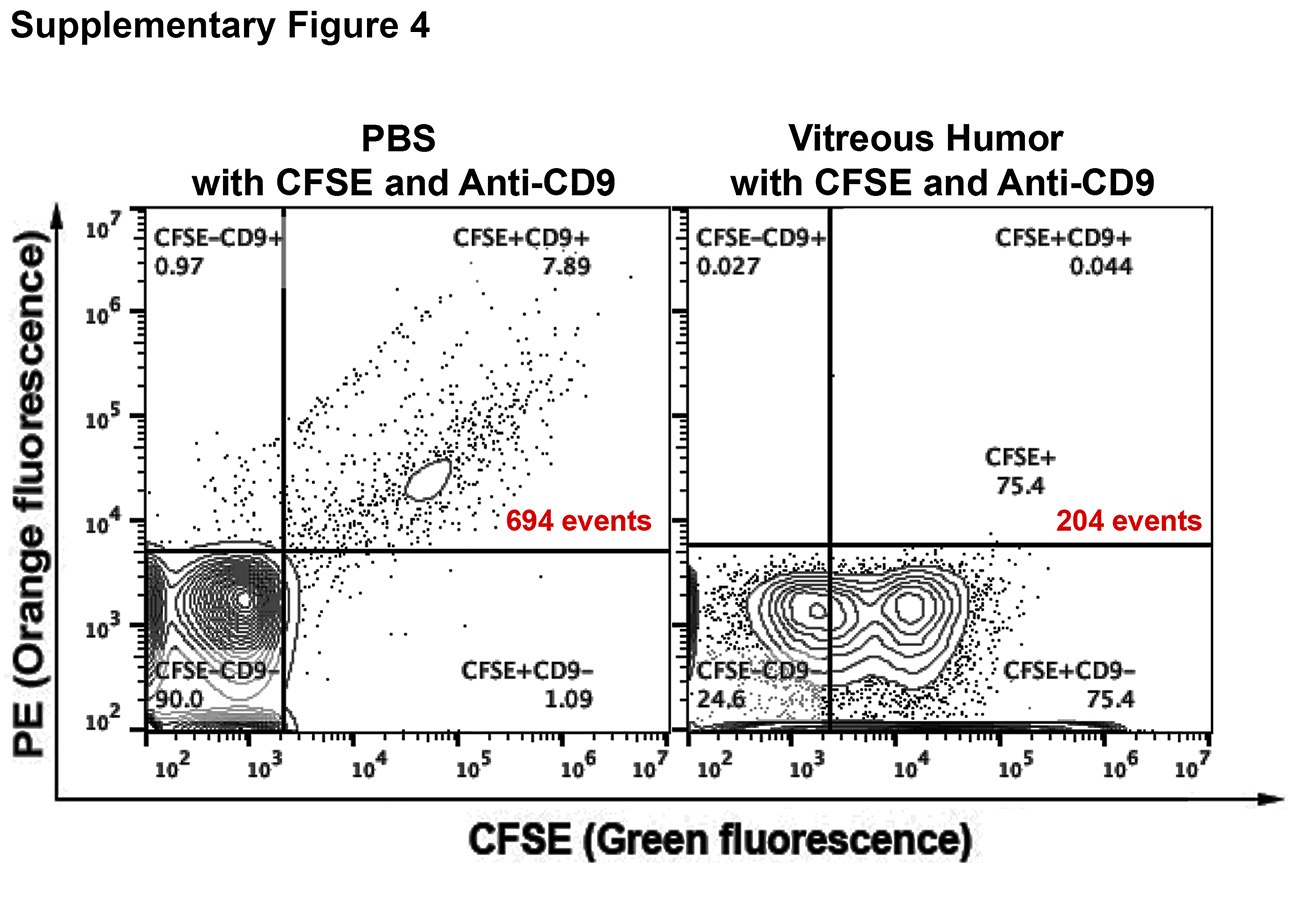

Supplement: Supplementary Figure 4 — CD9+ events in vitreous humor. Representative plots of PBS and vitreous humor double labeled with CFSE and anti-CD9. Samples were captured during 250 s. The indicated counts (CFSE+CD9+) correspond to the events within the upper right quadrant. [file Image_4.tif]

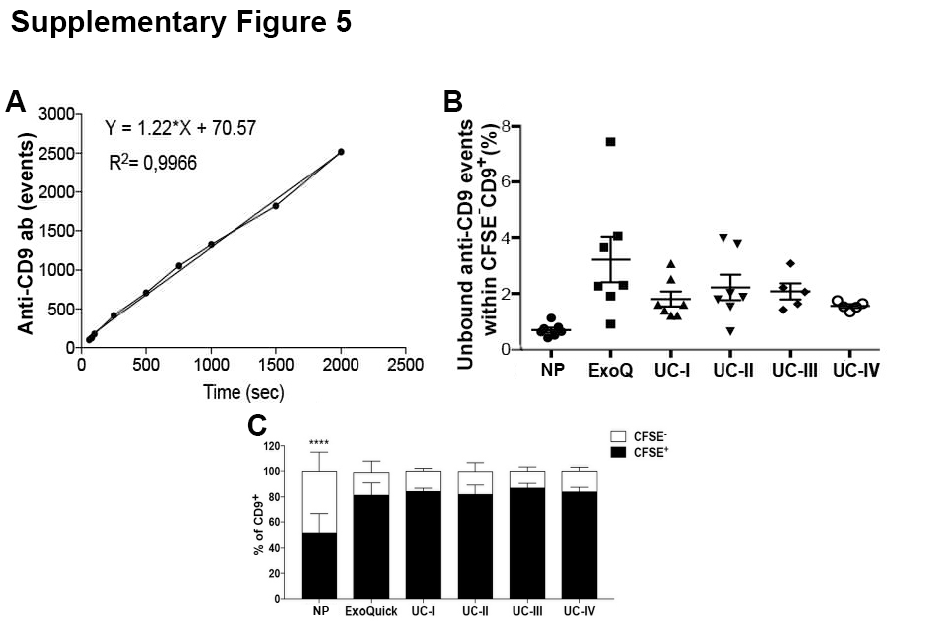

Supplement: Supplementary Figure 5 — Analysis of false-positive CD9+ events in conditioned medium. (A) Calibration curve of false-positive events. Buffer containing anti-CD9, in the same concentration used in the staining reaction and pre-cleared by SEC, was analyzed by Flow Cytometry for increasing acquisition times. (B) Estimate percentage of false-positive events by unbound anti-CD9 within CFSE–CD9+ in each purification context. (C) Proportion of CFSE+ (black) and CFSE– (white) events within CD9+ events in NP samples and samples purified by ExoQuick and ultracentrifugation (UC-I-IV). ****P < 0.0001 by ANOVA, with Tukey’s post-test. All data are represented as mean ±SEM. [file Image_5.tif]
